# Influence of Ocular Residual Astigmatism and Target-Induced Astigmatism on the Efficacy of the Implantation of a Toric Implantable Collamer Lens With Central Hole for Myopic Astigmatism Correction

**DOI:** 10.3389/fmed.2021.737358

**Published:** 2022-01-21

**Authors:** Jing Zhao, Jiao Zhao, Wen Yang, Ling Sun, Yishan Qian, Xiaoying Wang, Xingtao Zhou

**Affiliations:** ^1^Department of Ophthalmology, Eye and Ear, Nose, and Throat (ENT) Hospital of Fudan University, Shanghai, China; ^2^National Health Commission (NHC) Key Laboratory of Myopia, Laboratory of Myopia, Chinese Academy of Medical Sciences, Shanghai, China; ^3^Shanghai Research Center of Ophthalmology and Optometry, Shanghai, China; ^4^Department of Ophthalmology, People's Hospital of Leshan, Leshan, China; ^5^Department of Ophthalmology, The 3rd People's Hospital of Chengdu, Chengdu, China

**Keywords:** ocular residual astigmatism, target-induced astigmatism, myopic astigmatism, toric implantable collamer lens, corneal tomography

## Abstract

**Purpose:**

To investigate the effects of ocular residual astigmatism (ORA) and target-induced astigmatism (TIA) on the efficacy of toric implantable collamer lens (TICL) with central hole for myopic astigmatism correction.

**Methods:**

Retrospective case series. One hundred and eighteen eyes implanted with a TICL (V4c) from 118 patients were included. Subjective refraction and corneal topography were examined preoperatively, at 1 and 12 months postoperatively. The eyes were divided into the low-ORA ( ≤ 0.5 D) and high-ORA (>0.5 D) groups based on vector analysis, and into the low-TIA (≥0.75D and <2 D) and the high-TIA (≥2 D and ≤ 4 D) groups according to preoperative refractive astigmatism. Correction index (CI) and index of success (IOS) were compared between different groups.

**Results:**

All surgeries were uneventful, and no complications occurred during follow-up. At 1 and 12 months postoperatively, no significant differences were found in CI or IOS values between the high and low ORA groups, while significantly higher CI and lower IOS were detected in the high-TIA group than in the low-TIA group (*P* < 0.05). No significant difference was found in CI between 1 and 12 months postoperatively in either group (*P* > 0.05). However, significantly lower IOS was found at 12 months compared with 1 month postoperatively for each group (*P* < 0.05).

**Conclusions:**

Toric implantable collamer lens (TICL) implantation is effective in correcting myopic astigmatism and is more effective in eyes with high TIA, while ORA has a minor effect.

## Introduction

Ocular refractive astigmatism (RA) is a combination of corneal astigmatism (CA) and ocular residual astigmatism (ORA), CA representing the major component (1). However, the role of ORA should not be neglected. ORA can affect ocular astigmatism in different ways: it can partly neutralize CA, thus reducing ocular RA, or be superimposed with CA to aggravate RA (2). Grosvenor (3) reported constant against-the-rule astigmatism contribution from ORA to be close to 0.5 D.

Astigmatism can be corrected by refractive surgery including corneal laser treatment and intraocular lens implantation. Laser treatment has a variety of surgical methods, such as laser-assisted *in situ* keratomileusis (LASIK), laser-assisted subepithelial keratomileusis (LASEK), photorefractive keratectomy (PRK), and small incision lenticule extraction (SMILE). Laser correction of astigmatism has been mainly based on subjective refractive astigmatism. For eyes in which ORA is the main RA component, laser ablation might induce new astigmatism on the cornea, which is supposed to increase anterior corneal astigmatism to compensate for internal astigmatism. Our team (4–6) has demonstrated that ORA influences the efficacy of LASIK, LASEK, and SMILE in correcting myopic astigmatism when refractive correction is confined to the anterior cornea. Roszkowska (7) evaluated the efficacy, safety, stability, and predictability of PRK in correcting myopic astigmatism, hyperopic astigmatism, and mixed astigmatism, and demonstrated that PRK achieved satisfactory correction of all types of astigmatism with moderate and high cylinder magnitudes after 3 years of follow-up.

Safety, efficacy, and predictability of a toric implantable collamer lens (TICL) on myopic astigmatism correction have been reported (8, 9). Siedleck et al. (8) compared the effect of SMILE and ICL/TICL (V4c) on the correction of myopia or myopic astigmatism, finding that predictability of spherical equivalence (SE), uncorrected distance visual acuity (UDVA), higher-order aberration, and subjective visual quality of eyes implanted with ICL/TICL (V4c) were significantly better than those of patients who underwent SMILE. Wan et al. (9) found a significant difference in the correction index (CI) value between TICL implantation and SMILE for target-induced astigmatism (TIA) <2 D, but no significant differences for TIA ≥2 D.

The influence of ORA and TIA on myopic astigmatism correction by TICL (V4c) has not been specifically studied and remains unclear. In this study, we performed vector analysis to investigate the effects of ORA and TIA on the efficacy of TICL (V4c) for myopic astigmatism correction.

## Patients and Methods

### Patients

This retrospective study included patients who underwent routine preoperative examinations for TICL (V4c) implantation in the refractive surgery center of the Eye and ENT Hospital of Fudan University between August 2018 and June 2019.

Inclusion criteria were: spherical refraction −3 to −18 diopters (D), astigmatism 0.75–4 D, corrected distance visual acuity (CDVA) 20/40 or better, endothelium cell density (ECD) >2,000 cells/mm^2^, stable refraction for 2 years before surgery, and absence of other pathologic ocular conditions, of history of ocular trauma or surgery, and of systemic diseases.

This study adhered to the tenets of the Declaration of Helsinki, and approval was obtained from the Ethics Committee of the Eye and ENT Hospital of Fudan University. Written informed consent was obtained from each patient after the nature and possible consequences of the study were explained.

### TICL Calculation and Surgical Technique

Toric implantable collamer lens (TICL) sizing was based primarily on white-to-white distance and anterior chamber depth measurements, as recommended by the Staar surgical calculator (www.staarvision.com).

All surgeries were performed by two experienced surgeons using the same technique (Zhou and Wang). Standard TICL surgery was performed and a temporal 3-mm corneal incision was made, with the procedure described in our previous report (10). Binocular procedures were conducted successively, and the right eye was operated on first.

### Routine Examination

The patients were examined preoperatively and 1 and 12 months postoperatively. Uncorrected distance visual acuity (UDVA), CDVA, subjective refractive error, slit-lamp examination, intraocular pressure (IOP) measured with a tonometer (Canon Full Auto Tonometer TX-F; Canon, Inc., Tokyo, Japan), and ECD measured by non-contact specular microscopy (SP-2000P; Topcon Corporation, Japan) were recorded. Pentacam HR (Oculus Optikgeräte, Wetzlar, Germany) anterior segment examinations were performed by the same experienced examiner. Flat keratometry (Kf), steep keratometry (Ks), and the axis of Ks in central 3-mm diameter of three repeated measurements with “OK” quality were averaged for each result. Astigmatism of the anterior cornea in the central 3-mm ring in the positive-cylinder form is equal to the difference between Ks and Kf, with the same axis as Ks.

### Vector Analysis of Astigmatism

The right eyes were selected for analysis. The eyes were divided into the low-ORA (ORA ≤ 0.5 D) and high-ORA (ORA >0.5 D) group according to preoperative ORA values calculated by vector analysis; and into the low-TIA (≥0.75D and <2D) and high-TIA (≥2D and ≤ 4D) groups according to preoperative RA. ORA was determined as the vector difference between preoperative RA (corneal plane) and topographic (simulated keratometry) astigmatism (11, 12). The RA was converted into the corneal plane using a vertex of 12 mm. Because the target postoperative refraction was emmetropia in all the eyes, the magnitude of TIA was equal to that of the preoperative astigmatism and its axis perpendicular to that of the preoperative astigmatism.

Cylinder notation was decomposed into two cross-cylinder components by Fourier transformation (13). This provides J0 and J45 components that can be readily summed to determine corneal and intraocular contributions. The J0 and J45 components are defined as follows: J0 = –(C/2)^*^cos (2a); J45 = – (C/2)^*^sin (2a), where C is the cylinder power and a is the axis in radians. The J0 value represents the horizontal/vertical component of astigmatism, while J45 represents astigmatism in the 45° and 135° axes.

Astigmatic correction efficacy was assessed using a method established by Alpins (12). The index of success (IOS) is the ratio of uncorrected astigmatism (postoperative RA) to the initial preoperative RA. Higher IOS indicates higher proportion of preoperative RA uncorrected by TICL implantation. The SIA is the amount and axis of the astigmatic change caused by surgery, calculated as the vector difference between the pre- and postoperative astigmatism determined by corneal topography (12, 14). The CI is the ratio of surgical-induced astigmatism (SIA) to TIA. A CI value >1 (<1) means RA overcorrection (undercorrection).

### Statistical Analysis

Statistical analyses were performed using the SPSS 23.0 software (IBM, Armonk, NY, United States). Considering the importance of TIA and ORA, we independently analyzed the two different grouping methods that did not involve comparison between TIA and ORA. First, we examined the difference in sex by chi square test and baseline eye parameters by independent sample *t*-tests for normalized data or Mann-Whitney *U*-tests for unnormalized data across the TIA and ORA groups. Second, main effect analysis was performed for postoperative data by repeated measures ANOVA; if interested variables displayed significantly different at baseline, generalized estimation equation (GEE) was applied to adjust baseline value. Simple effect analysis was performed after main effect analysis, in which independent sample *t*-tests were conducted between the high- and low-ORA groups and between the high- and low-TIA groups, and paired *t* tests were implemented between postoperative 1- and 12-month for each group. Visual acuity was converted into the corresponding logarithm of the minimum angle of resolution (logMAR) value using standard conversion tables. *P*-values <0.05 were considered statistically significant.

## Results

### Preoperative Examinations and TICL Data

No significant differences were found in age, sex, sphere, RA, SE, CDVA, CA, TIA, IOP, and ECD between the high- and low-ORA groups (all *P* > 0.05). When comparing the low- and high-TIA groups, a significantly larger absolute value of RA, CA, and cylindrical power of TICL was found in the high-TIA group, while no significant differences were found in the other indices (*P* > 0.05; Table 1).

**Table 1:** Preoperative data stratified by ORA and TIA, respectively.

**Group**	**F/M**	**Age**	**Sphere**	**RA**	**SE**	**LogMAR CDVA**	**CA**	**ORA**	**cylindrical power of TICL**	**IOP**	**ECD**
Low ORA (*N* = 55)	35/20	28.16 ± 4.19 (20–37)	−9.42 ± 2.47 (−15 to −4.75)	−2.07 ± 0.97 (−4 to −1)	−10.46 ± 2.52 (−16 to −5.5)	0.02 ± 0.06 (−0.1 to 0.15)	1.80 ± 0.71 (0.5–3.3)	0.32 ± 0.10 (0.12–0.5)	2.07 ± 0.97 (1–4)	14.77 ± 2.61 (10–21)	2,833.36 ± 316.02 (2,118–3,715)
High ORA (*N* = 63)	38/35	28.59 ± 4.89 (19–38)	−8.93 ± 2.16 (−13.75 to −3.50)	−1.79 ± 0.78 (−4 to −1)	−9.84 ± 2.15 (−14.75 to −4.5)	0.00 ± 0.07 (−0.1 to 0.15)	1.88 ± 0.66 (0.3–3.4)	0.85 ± 0.27 (0.53–2.03)	1.79 ± 0.78 (1–4)	15.15 ± 2.30 (11–20)	2,789.81 ± 315.04 (2,085–3,798)
*P*-value	0.71	0.62	0.25	0.09	0.15	0.27	0.49	<0.001	0.09	0.39	0.46
Low TIA (*N* = 54)	25/20	27.85 ± 5.2 (19–38)	−9.23 ± 2.34 (−15 to −4.75)	−1.15 ± 0.23 (−1.5 to −1)	−9.81 ± 2.34 (−15.75 to −5.5)	0.00 ± 0.07 (−0.1 to 0.15)	1.35 ± 0.45 (0.3–2.8)	0.61 ± 0.35 (0.13–2.03)	1.15 ± 0.23 (1–1.5)	14.86 ± 2.24 (10–21)	2,776.93 ± 270.55 (2,214–3,450)
High TIA (*N* = 64)	39/34	28.84 ± 3.93 (22–37)	−9.10 ± 2.31 (−15 to −3.50)	−2.58 ± 0.67 (−4 to −2)	−10.39 ± 2.32 (−16 to −4.5)	0.01 ± 0.07 (−0.1 to 0.15)	2.25 ± 0.54 (1–3.4)	0.60 ± 0.33 (0.12–1.36)	2.58 ± 0.66 (2–4)	15.07 ± 2.62 (10–20)	2,838.11 ± 347.62 (2,085–3,798)
*P*–value	0.821	0.25	0.76	<0.001	0.18	0.69	<0.001	0.94	<0.001	0.64	0.30

*F, female; M, male; SE, spherical equivalence; RA, refractive astigmatism by manifest refraction; CA, corneal astigmatism; ORA, ocular residual astigmatism; LogMAR, log minimum angle of resolution; CDVA, corrected distance visual acuity; IOP, intraocular rressure; ECD, endothelial cell density; TIA, target induced astigmatism (equal to refractive astigmatism by manifest refraction)*.

### Safety and Efficacy

All the surgeries were uneventful, and no complications occurred during follow-up. At 12 months postoperatively, no significant difference was found in UDVA (−0.07 ± 0.07 vs. −0.08 ± 0.07) or CDVA (−0.10 ± 0.07 vs. −0.1 ± 0.07) between the low- and high-ORA groups, while significant difference in UDVA was found between the low- and high-TIA groups (−0.09 ± 0.07 vs. −0.05 ± 0.06), but there was no clinical significance.

**Figure 1:**
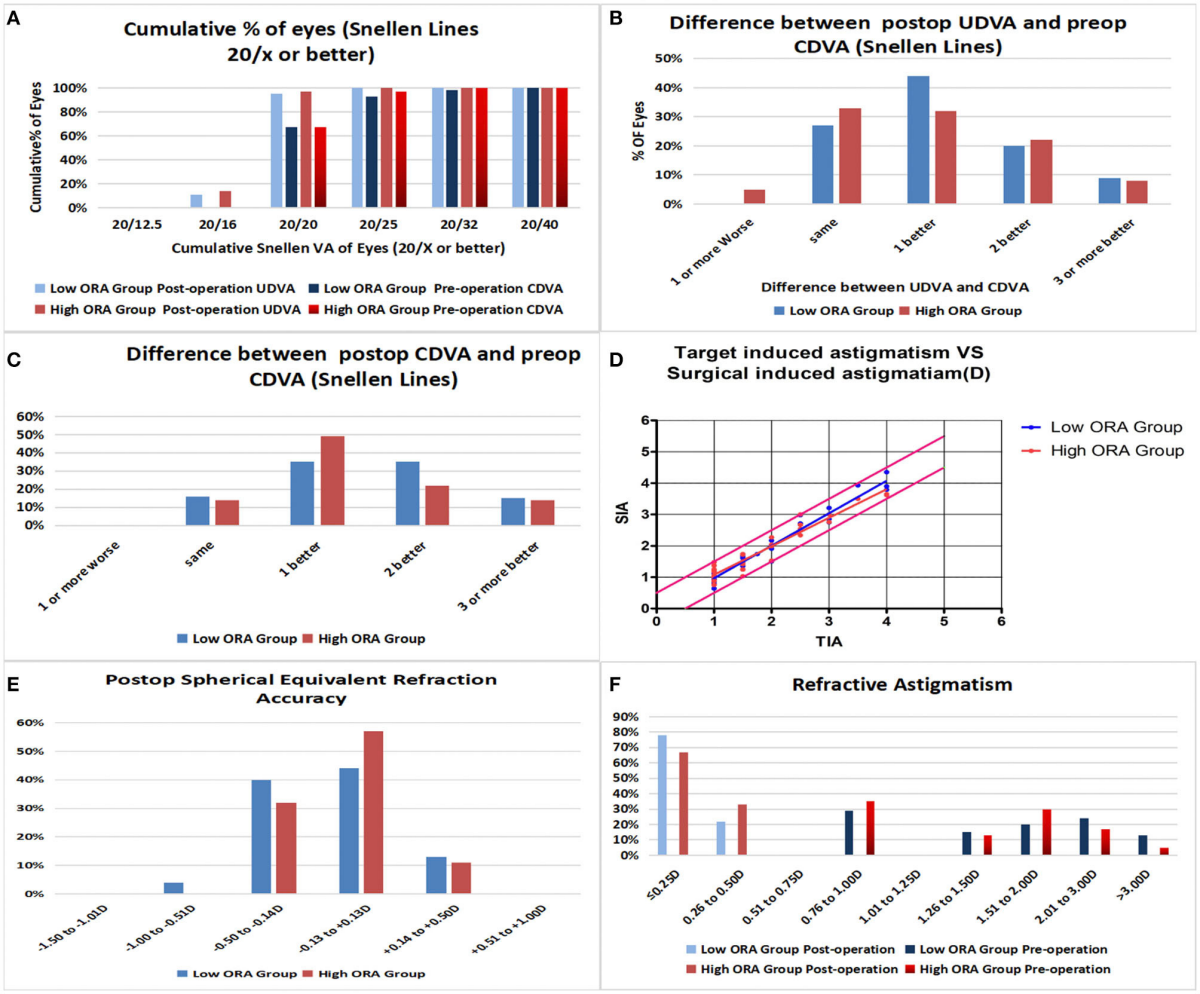
Refractive outcomes in the low- and high- ocular residual astigmatism (ORA) groups. **(A)** Cumulative percentage of eyes attaining specified levels of uncorrected distance visual acuity (UDVA); **(B)** Postoperative vs. preoperative UDVA; **(C)** Change in corrected distance visual acuity (CDVA); **(D)** Target-induced astigmatism plotted vs. surgical-induced astigmatism at the last follow-up; **(E)** Distribution of postoperative spherical equivalent refraction; **(F)** Distribution of preoperative and postoperative astigmatism amplitudes.

**Figure 2:**
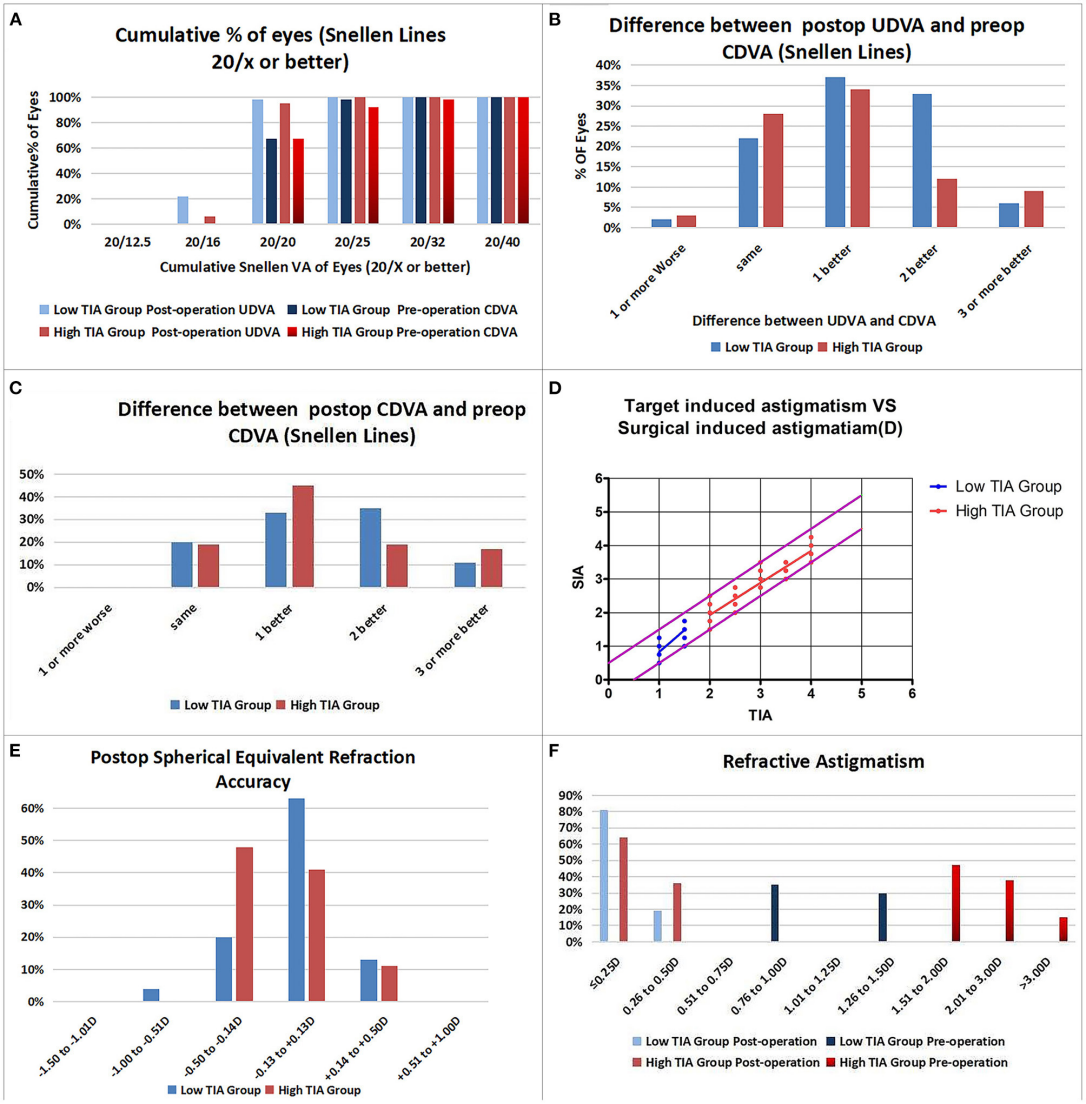
Refractive outcomes in the low- and high-target-induced astigmatism (TIA) groups. **(A)** Cumulative percentage of eyes attaining specified levels of UDVA; **(B)** Postoperative vs. preoperative UDVA; **(C)** Change in CDVA; **(D)** TIA plotted vs. surgical-induced astigmatism at the last follow-up; **(E)** Distribution of postoperative spherical equivalent refraction; **(F)** Distribution of preoperative and postoperative astigmatism amplitudes.

Efficacy indices (postoperative UDVA/preoperative CDVA) were 1.2 ± 0.18 and 1.17 ± 0.19 for the low- and high-ORA groups, respectively, at 12 months postoperatively. All the eyes in both groups had postoperative UDVA ≥20/40, and 95 and 97% of the eyes in the low- and high-ORA groups achieved better than 20/20 (Figure 1A). However, the UDVA in three eyes (5%) declined by one line compared with the preoperative CDVA in the high-ORA group (Figure 1B). In the low- and high-TIA groups, efficacy indices were 1.23 ± 0.19 and 1.15 ± 0.17, respectively. All the eyes in both groups had postoperative UDVA ≥20/40, and 98 and 95% of the eyes in the low- and high-TIA groups, respectively, achieved better than 20/20 (Figure 2A). The UDVA in one eye (2%) and two eyes (3%) declined by one line compared with the preoperative CDVA in the low- and high-TIA groups, respectively (Figure 2B). No significant differences were found in safety or efficacy indices between groups stratified by ORA or TIA (*P* > 0.05).

In the low-ORA group, the safety index (postoperative/preoperative CDVA) was 1.29 ± 0.2. In 16% of the eyes, the preoperative CDVA was maintained, in 84%, it increased by one or more lines, in 50% by two or more, and no CDVA declined. In the high-ORA group, the safety index was 1.26 ± 0.18. In 14% of eyes, the preoperative CDVA was maintained, in 86%, it increased by one or more lines, in 36% by two, and no CDVA declined (Figure 1C). In the low-TIA group, the safety index was 1.26 ± 0.19. In 20% of the eyes, the preoperative CDVA was maintained, in 80%, it increased by one or more lines, in 46% by two, and no CDVA declined. In the high-TIA group, the safety index was 1.26 ± 0.23. In 19% of the eyes, the preoperative CDVA was maintained, in 81%, it increased by one or more lines, in 36% by two or more, and no CDVA declined (Figure 2C).

### Predictability

At 12 months postoperatively, 53 eyes (96%) in the low-ORA group achieved within ± 0.5D of the attempted SE, and 78% achieved postoperative astigmatism within 0.25D. All the 63 eyes in the high-ORA group achieved within ± 0.5D of the attempted SE, and 67% achieved postoperative astigmatism within 0.25D; all the eyes had postoperative astigmatism within 0.5D in both groups (Figures 1D–F).

In the low-TIA group, 52 eyes (96%) achieved within ± 0.5D of the attempted SE, and 81% achieved postoperative astigmatism within 0.25D. In the high-TIA group, all 64 eyes achieved within ±0.50 D of the attempted SE, and 64% achieved postoperative astigmatism within 0.25D; all the eyes had postoperative astigmatism within 0.5D in both groups (Figures 2D–F).

### Other Ocular Measurements

There was no rotation of TICL in all cases. No significant differences were found in vault, IOP, or ECD at 12 months postoperatively between the groups stratified by ORA or TIA (*P* > 0.05; Tables 2, 3). Vault decreased by 4.94 ± 7.53, 7.06 ± 9.73, 5.07 ± 7.08, and 7.39 ± 9.95 μm at 12 months postoperatively compared with 1 month postoperatively for the low-ORA, high-ORA, low-TIA, and high-TIA groups, respectively. The ECD value in the corresponding groups at 12 months postoperatively was 2,817.73 ± 317.37, 2,773.14 ± 314.15, 2,762.39 ± 268.3, and 2,820.53 ± 349.64 cells/mm^2^, respectively, corresponding to a 0.56, 0.58, 0.57, and 0.59% decrease compared with preoperative values.

**Table 2:** Postoperative comparison of low ORA group and high ORA group.

	**Low ORA group (*****N*** **=** **53)**		**High ORA group (*****N*** **=** **65)**		**Group**	**Time**	Group^*^time
	**1 m**	**12 m**	**1 m**	**12 m**	***P***	***P***	***P***
LogMar UDVA	−0.05 ± 0.07	−0.07 ± 0.07	−0.07 ± 0.07	−0.08 ± 0.07	0.87	0.69	0.10
LogMar CDVA	−0.07 ± 0.07^a^^c^	−0.10 ± 0.07^c^	−0.10 ± 0.07^a^	−0.10 ± 0.07	0.24	0.01	<0.001
SE(D)	−0.08 ± 0.26	−0.11 ± 0.24	−0.04 ± 0.26^d^	−0.07 ± 0.22^d^	0.43	0.08	0.85
Cylinder diopter by MR(D)	−0.32 ± 0.18^c^	−0.26 ± 0.16^c^	−0.34 ± 0.20^d^	−0.28 ± 0.18^d^	0.52	0.01	0.83
CA(D)	2.00 ± 0.73	1.97 ± 0.80	2.00 ± 0.72	1.95 ± 0.72	0.93	0.31	0.49
Vault (μm)	529.13 ± 191.91^c^	524.18 ± 192.12^c^	525.63 ± 148.53^d^	518.57 ± 148.78^d^	0.91	<0.001	0.16
IOP	14.88 ± 2.50	14.75 ± 2.64	15.31 ± 2.24	15.23 ± 2.43	0.25	0.15	0.69
Refractive SIA	1.90 ± 0.99	1.98 ± 0.98	1.66 ± 0.85	1.67 ± 0.84	0.14	0.63	0.93
Corneal SIA	0.35 ± 0.25	0.31 ± 0.25	0.35 ± 0.20	0.35 ± 0.26	0.53	0.70	0.34
CI	0.89 ± 0.17	0.94 ± 0.14	0.90 ± 0.19	0.91 ± 0.14	0.76	0.38	0.22
IOS	0.19 ± 0.14^c^	0.15 ± 0.10^c^	0.22 ± 0.16^d^	0.19 ± 0.15^d^	0.19	0.02	0.94

*ORA, ocular residual astigmatism; CDVA, corrected distance visual acuity; UDVA, uncorrected distance visual acuity; SE, spherical equivalence; MR, manifest refraction; SIA, surgical induced astigmatism; CA, corneal astigmatism; IOP, intraocular pressure; IOS, index of success; CI, correction index*.

a *The low ORA group at post 1 month vs. the high ORA group at post 1 month*;

b *the low ORA group at post 12 months vs. the high ORA group at post 12 months*;

c *the low ORA group at post 1 month vs. the low ORA group at post 12 months*;

d *the high ORA group at post 1 month vs. the high ORA group at post 12 months*.

* *Stands for interaction between Group and Time*.

**Table 3:** Postoperative comparison of low and high TIA Group.

	**Low TIA group (*****N*** **=** **54)**		**High TIA Group (*****N*** **=** **64)**		**Group**	**Time**	Group*time
	**1 m**	**12 m**	**1 m**	**12 m**	***P***	***P***	***P***
LogMarUDVA	−0.08 ± 0.07^a^	−0.09 ± 0.07^b^	−0.05 ± 0.06^a^	−0.05 ± 0.06^b^	0.003	0.14	0.45
LogMarCDVA	−0.11 ± 0.07	−0.11 ± 0.07	−0.09 ± 0.06	−0.10 ± 0.06	0.12	0.11	0.11
SE(D)	−0.05 ± 0.25	−0.05 ± 0.22^b^	−0.05 ± 0.27^d^	−0.12 ± 0.23^bd^	0.46	0.006	0.003
Cylinder diopter by MR(D)	−0.31 ± 0.17^c^	−0.26 ± 0.15^c^	−0.36 ± 0.20^d^	−0.29 ± 0.19^d^	0.21	<0.001	0.30
CA(D)	1.49 ± 0.48^a^^c^	1.43 ± 0.47^b^^c^	2.43 ± 0.61^a^	2.40 ± 0.66^b^	<0.001	0.02	0.40
Vault (μm)	538.41 ± 176.71^c^	534.07 ± 176.59^c^	517.86 ± 163.77^d^	510.31 ± 164.13^d^	0.48	<0.001	0.48
IOP	15.08 ± 2.14	14.97 ± 2.39	15.14 ± 2.56	15.04 ± 2.66	0.89	0.26	0.93
Refractive SIA	1.00 ± 0.37^a^	1.02 ± 0.35^b^	2.42 ± 0.72^a^^d^	2.48 ± 0.68^b^^d^	<0.001	0.025	0.38
Corneal SIA	0.30 ± 0.16^a^	0.27 ± 0.21^b^	0.39 ± 0.26^a^	0.39 ± 0.28^b^	0.004	0.42	0.62
CI	0.85 ± 0.23^a^	0.88 ± 0.17^b^	0.93 ± 0.10^a^	0.96 ± 0.09^b^	0.001	0.07	0.77
IOS	0.28 ± 0.18^a^^c^	0.23 ± 0.14^b^^c^	0.15 ± 0.10^a^^d^	0.12 ± 0.09^b^^d^	<0.001	<0.001	0.43

*TIA, target induced astigmatism; CDVA, corrected distance visual acuity; UDVA, uncorrected distance visual acuity; SE, spherical equivalence; MR, manifest refraction; CA, corneal astigmatism; SIA, surgical induced astigmatism; IOP, intraocular pressure; IOS, index of success; CI, correction index*.

a *The low TIA group at post 1 month vs. the high TIA group at post 1 month*;

b *the low TIA group at post 12 months vs. the high TIA group at post 12 months*;

c *the low TIA group at post 1 month vs. the low TIA group at post 12 months*;

d the high TIA group at post 1 month vs. the high TIA group at post 12 months. *Stands for interaction between Group and Time.

### CI and IOS in the Low- and High-ORA Groups

**Figure 3:**
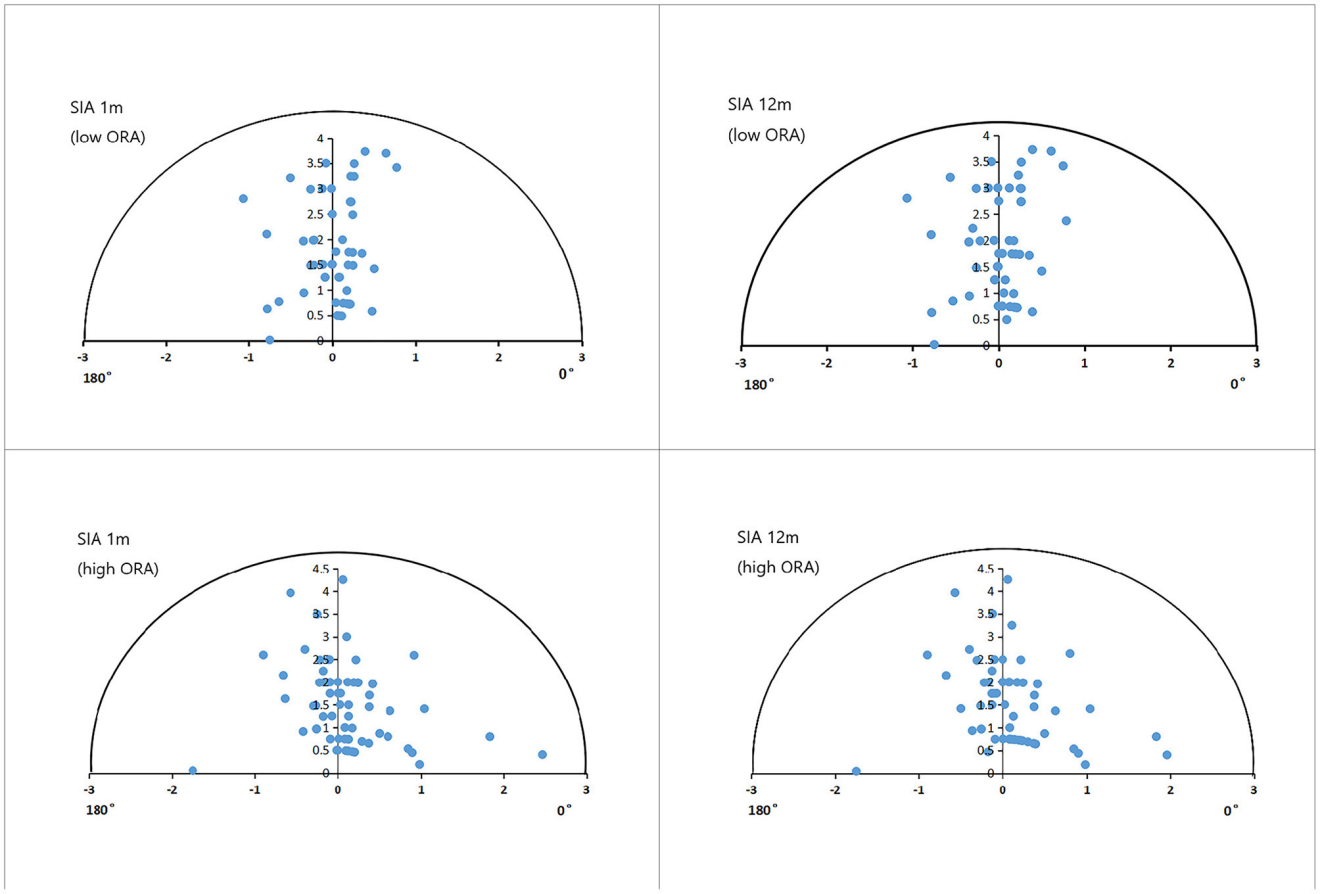
Single-angle polar plots of refractive SIA in the low- and high-ORA groups. ORA, ocular residual astigmatism; SIA, surgical-induced astigmatism.

Figure 3 shows the refractive SIA on polar diagrams in the low- and high-ORA groups at 1 and 12 months postoperatively. There were no significant differences in refractive or corneal SIA between the two groups at any postoperative timepoint, or between timepoints in any group (*P* > 0.05). No significant differences were found in CI or IOS between the low- and high-ORA groups at the same timepoints (*P* > 0.05), or in the CI between the two postoperative timepoints for either group (*P* > 0.05). However, a significantly lower IOS was found at 12 months compared with 1 month postoperatively in both groups (*P* < 0.05; Table 2).

### CI and IOS in the Low- and High-TIA Groups

**Figure 4:**
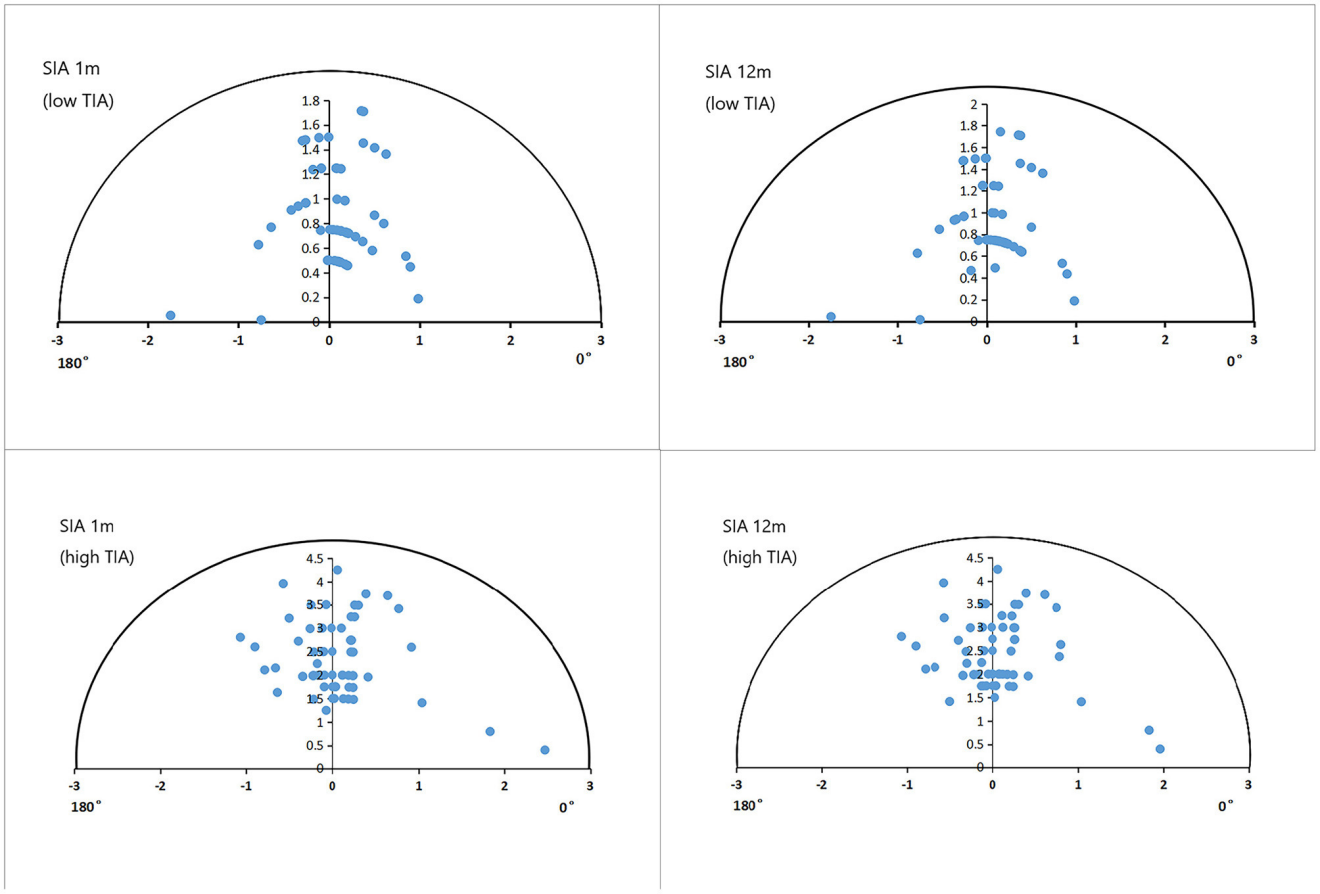
Single-angle polar plots of refractive SIA in the low- and high-TIA groups. ORA, ocular residual astigmatism; SIA, surgical-induced astigmatism; TIA, target-induced astigmatism.

Figure 4 shows the refractive SIA on polar diagrams in the low- and high-TIA groups at 1 and 12 months postoperatively. Both refractive and corneal SIA values were smaller in the low- than in the high-TIA group at both timepoints (*P* < 0.05). There were no significant differences in corneal SIA between the two timepoints in either group (*P* > 0.05). At 1 and 12 months postoperatively, significantly higher CI and lower IOS were found in the high- compared with the low-TIA group (*P* < 0.05). No significant CI differences were found between the two postoperative timepoints for either group (*P* > 0.05). However, significantly lower IOS was found at 12 months compared with 1 month postoperatively for both groups (*P* < 0.05) (Table 3).

## Discussion

Astigmatism has always been a concern in refractive surgery. Although an astigmatic residue of 0.5D has no obvious effect on vision, it does affect the visual quality of patients (15). Therefore, accurate correction of astigmatism is of utmost importance for TICL implantation and corneal refractive surgery. Recently, TICL (V4c) implantation has attracted increasing attention because of its reversibility, safety, effectiveness, excellent predictability, and postoperative visual quality (16, 17). However, whether ORA or TIA affects the efficacy of TICL (V4c) in correcting astigmatism has not been reported. We explored this question for the first time in this study.

In this study, the safety and efficacy indices of the operated eyes were within a satisfactory range, in accordance with prior studies on TICL (V4) by Kamiya et al. (18) and on ICL (V4c) by our team (19). In this study, postoperative UDVA in three eyes (2.54%) declined by one line compared with preoperative CDVA, as the target refraction with high myopia was more inclined to a postoperative shift toward myopia. Similar to the report by Garcia-De la Rosa et al. (20), the vault slightly decreased, within the safe range at 12 months postoperatively. The ECD decreased by approximately 0.6% after 1 year, in accordance with physiological loss (21). This study further demonstrated the excellent safety and efficacy of TICL (V4c) implantation for astigmatism correction in myopia. The increased retinal image magnification and reduced spot size with TICL (V4c) in the posterior chamber might account for postoperative visual improvement (22).

Sari et al. (23) reported that the mean cylindrical power was 0.49D 3 years after TICL (V4) implantation. De la Rosa et al. (20) reported a decrease from 2.87D preoperatively to 0.28D 12 months postoperatively, and 78% of the eyes were within 1D. Although the mean astigmatic diopters were similar between the report by De la Rosa and this study, the predictability of cylindrical diopters in this study was better than that reported by De la Rosa et al., possibly because of (1) the maximum astigmatic diopter of eyes (4D in this study vs. 7D in De la Rosa et al.) for the small range of RA of TICL implanted patients during the observation time, and (2) accuracy of the TICL axial placement and difference in its rotation stability.

In this study, the efficacy of astigmatic correction was analyzed by Alpins vector analysis (14). When stratified by ORA, the CI for both the low- and high-ORA groups was <1 at 1 and 12 months postoperatively, indicating astigmatic undercorrection, which was also detected in prior studies on different models of TICLs and corneal refractive surgery (18, 23, 24). For corneal refractive surgery, nomogram adjustment for tissue-saving ablation profile for the correction of high myopia and astigmatism might result in undercorrection. In TICL implantation, astigmatic undercorrection might be related to the fact that the corneal SIA is not taken into consideration in the online calculation formula currently used. Corneal SIA was reported to be 0.59 and 0.45D for femtosecond laser and manual clear corneal incisions, respectively (25). The mean corneal SIA with 3-mm clear corneal incisions was 0.33D at 12 months postoperatively in this study. Therefore, it is necessary to take the corneal SIA into account to achieve accurate astigmatism correction through TICL implantation.

The efficacy of astigmatic correction was compared between the low- and high-ORA groups in this study, and no significant differences were found in refractive SIA, corneal SIA, CI, or IOS. In our previous study on the influence of ORA on the correction of myopic astigmatism by SMILE 6 months postoperatively, there was a significant difference in mean IOS between the two groups (high-ORA: 0.77; low-ORA: 0.46) (4). In another study focused on LASEK, we found the mean IOS to be 0.88 and 0.32 in the high- and low-ORA groups, respectively, 3 months postoperatively (*P* = 0.04) (6). Our prior studies indicated that corneal refractive surgery was less effective in correcting mainly intraocular myopic astigmatism, and that ORA should be considered in surgical planning in addition to manifest astigmatism. The discrepancy in the effect of ORA on astigmatic correction by TICL and corneal refractive surgery might be due to the fact that TICL implantation does not involve corneal ablation, thus having less influence on the effect of astigmatic correction.

When the eyes were stratified by TIA, our results were in accordance with those of previous studies on the efficacy of astigmatic correction by corneal refractive surgery and TICL implantation. At 12 months postoperatively, the CI was significantly higher, and the IOS was significantly lower in the high- than in the low-TIA group, indicating higher efficacy of TICL (V4c) in correcting high astigmatism compared with low astigmatism. Wan et al. (9) divided the eyes into the <2D and ≥2D groups based on the TICL cylindrical power, finding significantly higher CI and significantly lower IOS in the ≥2D group. Our prior study on the efficacy of SMILE in correcting astigmatism found that with TIA <0.5D, there was no significant difference in RA between pre- and postoperative timepoints (6 months postoperatively vs. preoperatively: 0.37 ± 0.34D vs. 0.34 ± 0.17D) (4). When TIA was between 0.5 and 1D, the postoperative RA was significantly lower than the preoperative value (6 months postoperatively vs. preoperatively: 0.46 ± 0.39D vs. 0.87 ± 0.13D) (4). The results of this study were consistent with previous studies on the correction of astigmatism by TICL (V4c) and SMILE surgery, indicating that in both corneal refractive surgery and intraocular lens implantation, the efficacy of astigmatic correction in eyes with high TIA is better than in those with low TIA. The better sensitivity and accuracy of the axial position, and the large magnitude of astigmatism in optometry might account for this difference.

Whether stratified by ORA or TIA, no significant differences were found in corneal SIA or CI value between 1 and 12 months postoperatively, indicating that the SIA stabilized after 1 month. However, there was a statistically significant difference in RA and IOS values between the two postoperative timepoints in all the groups, with declining trend in absolute values. As the age-related change in astigmatism is known to be <0.25 diopters (D)/10 years (26, 27), the cause needs further investigation. In addition, the mean postoperative RA for all eyes at 1 and 12 months was 0.33 and 0.27 D, respectively. Astigmatism lower than 0.5D has little impact on visual acuity and might not need correction in clinical practice (15). Therefore, we believe that the difference in postoperative astigmatism at 1 and 12 months after surgery had little clinical significance.

Our study has some limitations. First, the follow-up time was relatively short. Second, the range of astigmatic diopters was small. Future studies should include a wider range of astigmatic diopters, such as from 0.5 to 6D, for a longer period using prospective research methods.

In conclusion, TICL (V4c) implantation is effective in correcting myopic astigmatism mainly at the internal optics and is more effective in correcting eyes with high TIA than those with low TIA. In addition, ORA has a minor effect on astigmatism correction through TICL (v4c) implantation.

## Data Availability Statement

The original contributions presented in the study are included in the article/supplementary material, further inquiries can be directed to the corresponding author/s.

## Ethics Statement

The studies involving human participants were reviewed and approved by the Ethics Committee of the Eye and ENT Hospital of Fudan University. The patients/participants provided their written informed consent to participate in this study.

## Author Contributions

The study concept, design were formulated, drafting of the manuscript was carried out, and critical revision of the manuscript was done by JinZ, JiaZ, and XZ. Data collection was done by JinZ, JiaZ, WY, LS, XW, and XZ. Analysis and interpretation of data was undertaken by JinZ, JiaZ, YQ, and XZ. Supervision was done by XZ. All authors contributed to the article and approved the submitted version.

## Funding

This study was supported by the National Natural Science Foundation of China (Grant No: 81770955), joint research project of new frontier technology in municipal hospitals (SHDC12018103), Project of Shanghai Science and Technology (Grant No: 20410710100), Clinical Research Plan of SHDC (SHDC2020CR1043B), and Project of Shanghai Xuhui District Science and Technology (2020-015).

## Conflict of Interest

The authors declare that the research was conducted in the absence of any commercial or financial relationships that could be construed as a potential conflict of interest.

## Publisher's Note

All claims expressed in this article are solely those of the authors and do not necessarily represent those of their affiliated organizations, or those of the publisher, the editors and the reviewers. Any product that may be evaluated in this article, or claim that may be made by its manufacturer, is not guaranteed or endorsed by the publisher.

## References

[1] Lin J. The contribution of ocular residual astigmatism to anterior corneal astigmatism in refractive astigmatism eyes. Sci Rep. (2021) 11:1018. 10.1038/s41598-020-80106-6 33441809 PMC7806660

[2] Harvey EM Miller JM Twelker JD Sherrill DL. Longitudinal change and stability of refractive, keratometric, and internal astigmatism in childhood. Invest Ophthalmol Vis Sci. (2014) 56:190–98. 10.1167/iovs.14-13898 25515577 PMC4290560

[3] Grosvenor T Quintero S Perrigin DM. Predicting refractive astigmatism: a suggested simplification of Javal's rule. Am J Optom Physiol Opt. (1988) 65:292–7. 10.1097/00006324-198804000-00009 3377064

[4] Qian Y Huang J Chu R Zhao J Li M Zhou X. Influence of intraocular astigmatism on the correction of myopic astigmatism by femtosecond laser small-incision lenticule extraction. J Cataract Refract Surg. (2015) 41:1057–64. 10.1016/j.jcrs.2014.09.036 26049837

[5] Qian Y Huang J Zhou X Wang Y. Comparison of femtosecond laser small-incision lenticule extraction and laser-assisted subepithelial keratectomy to correct myopic astigmatism. J Cataract Refract Surg. (2015) 41:2476–86. 10.1016/j.jcrs.2015.05.043 26703499

[6] Qian Y Huang J Chu R Zhou X Olszewski E. Influence of intraocular astigmatism on the correction of myopic astigmatism by laser-assisted subepithelial keratectomy. J Cataract Refract Surg. (2014) 40:558–63. 10.1016/j.jcrs.2013.09.017 24581997

[7] Roszkowska AM De Grazia L Meduri A Wylegala E Aragona P. Long-term results of excimer laser procedure to correct astigmatic refractive errors. Med Sci Monit. (2013) 19:927–33. 10.12659/MSM.884023 24185613 PMC3829742

[8] Siedlecki J Schmelter V Mayer WJ Schworm B Priglinger SG Dirisamer M. Luft smile versus implantable collamer lens implantation for high myopia: a matched comparative study. J Refract Surg. (2020) 36:150–9. 10.3928/1081597X-20200210-02 32159819

[9] Wan T Yin H Wu Z Yang Y. Vector analysis of small incision lenticule extraction and toric implantable collamer lens implantation for astigmatism correction. Eur J Ophthalmol. (2020) 2020:1–8. 10.1177/1120672120930607 32468871

[10] Zhao J Luo D Sun Y Niu L Zhao F Wang X. Implanting a posterior chamber phakic intraocular lens in highly myopic eyes with peripheral primary iris and ciliary body cysts. Eur J Ophthalmol. (2019) 29:171–7. 10.1177/1120672118766445 29607656

[11] Holladay JT Moran JR Kezirian GM. Analysis of aggregate surgically induced refractive change, prediction error, and intraocular astigmatism. J Cataract Refract Surg. (2001) 27:61–79. 10.1016/S0886-3350(00)00796-3 11165858

[12] Alpins N. Astigmatism analysis by the Alpin smethod. J Cataract Refract Surg. (2001) 27:31–49. 10.1016/S0886-3350(00)00798-7 11165856

[13] Thibos LN Wheeler W Horner D. Power vectors: an application of fourier analysis to the description and statistical analysis of refractive error. Optom Vis Sci. (1997) 74:367–75. 10.1097/00006324-199706000-00019 9255814

[14] Alpins NA. New method of targeting vectors to treat astigmatism. J Cataract Refract Surg. (1997) 23:65–75. 10.1016/S0886-3350(97)80153-8 9100110

[15] Villegas EA Alcón E Artal P. Minimum amount of astigmatism that should be corrected. J Cataract Refract Surg. (2014) 40:13–9. 10.1016/j.jcrs.2013.09.010 24355718

[16] Sanders DR Doney K Poco Poco M; ICL in Treatment of Myopia Study Group. United States food and drug administration clinical trial of the implantable collamer lens (ICL) for moderate to high myopia: three-year follow-up. Ophthalmology. (2004) 111:1683–92. 10.1016/j.ophtha.2004.03.026 15350323

[17] Schallhorn S Tanzer D Sanders DR Sanders ML. Randomized prospective comparison of visian toric implantable collamer lens and conventional photorefractive keratectomy for moderate to high myopic astigmatism. J Refract Surg. (2007) 23:853–67. 10.3928/1081-597X-20071101-01 18041238

[18] Kamiya K Shimizu K Aizawa D Igarashi A Komatsu M Nakamura A. One-year follow-up of posterior chamber toric phakic intraocular lens implantation for moderate to high myopic astigmatism. Ophthalmology. (2010) 117:2287–94. 10.1016/j.ophtha.2010.03.054 20598749

[19] Chen X Guo L Han T Wu L Wang X Zhou X. Contralateral eye comparison of the long-term visual quality and stability between implantable collamer lens and laser refractive surgery for myopia. Acta Ophthalmol. (2019) 97:e471–8. 10.1111/aos.13846 30187653 PMC6585688

[20] Garcia-De la Rosa G Olivo-Payne A Serna-Ojeda JC Salazar-Ramos MS Lichtinger A Gomez-Bastar A. Anterior segment optical coherence tomography angle and vault analysis after toric and non-toric implantable collamer lens V4c implantation in patients with high myopia. Br J Ophthalmol. (2018) 102:544–8. 10.1136/bjophthalmol-2017-310518 28729370

[21] Bourne WM Nelson LR Hodge DO. Central corneal endothelial cell changes over a ten-year period. Invest Ophthalmol Vis Sci. (1997) 38:779–82. 9071233

[22] Alió JL Ortiz D Abdelrahman A de Luca A. Optical analysis of visual improvement after correction of anisometropic amblyopia with a phakic intraocular lens in adult patients. Ophthalmology. (2007) 114:643–7. 10.1016/j.ophtha.2006.07.053 17188361

[23] Sari ES Pinero DP Kubaloglu A Evcili PS Koytak A Kutlutürk I. Toric implantable collamer lens for moderate to high myopic astigmatism: 3-year follow-up. Graefes Arch Clin Exp Ophthalmol. (2013) 251:1413–22. 10.1007/s00417-012-2172-8 23052720

[24] Pedersen IB Ivarsen A Hjortdal J. Changes in astigmatism, densitometry, and aberrations after smile for low to high myopic astigmatism: a 12-month prospective study. J Refract Surg. (2017) 33:11–7. 10.3928/1081597X-20161006-04 28068441

[25] Zhu S Qu N Wang W Zhu Y Shentu X Chen P. Morphologic features and surgically induced astigmatism of femtosecond laser versus manual clear corneal incisions. J Cataract Refract Surg. (2017) 43:1430–5. 10.1016/j.jcrs.2017.08.011 29223232

[26] Shao X Zhou KJ Pan AP Cheng XY Cai HX Huang JH. Age-related changes in corneal astigmatism. J Refract Surg. (2017) 33:696–703. 10.3928/1081597X-20170718-04 28991338

[27] Naeser K Savini G Bregnhøj JF. Age-related changes in with-the-rule and oblique corneal astigmatism. Acta Ophthalmol. (2018) 96:600–6. 10.1111/aos.13683 29369508

